# Advanced glycation end products and their ratio to soluble receptor are associated with limitations in physical functioning only in women: results from the CARLA cohort

**DOI:** 10.1186/s12877-019-1323-8

**Published:** 2019-11-04

**Authors:** Helen Ebert, Maria Elena Lacruz, Alexander Kluttig, Andreas Simm, Karin Halina Greiser, Daniel Tiller, Nadja Kartschmit, Rafael Mikolajczyk

**Affiliations:** 1 Institute of medical epidemiology, biometrics and informatics, Medical faculty of the Martin-Luther University Halle, Magdeburger Str. 8, 06112 Halle, Germany; 2 University Clinic and Outpatient Clinic for Cardiac Surgery,Middle German Heart Centre at the University Hospital Halle, Halle, Germany; 3 German Cancer Research Center, Division of Cancer Epidemiology, Heidelberg, Germany

**Keywords:** Advanced glycosylation, Physical function, Biomarker, Disability

## Abstract

**Background:**

Advanced glycation end products (AGEs), modifications of proteins or amino acids, are increasingly produced and accumulated with age-related diseases. Recent studies suggested that the ratio of AGEs and their soluble receptor (sRAGE) is a more accurate biomarker for age-related diseases than each separately. We aim to investigate whether this also applies for physical functioning in a broad age-spectrum.

**Methods:**

AGE and sRAGE levels, and physical functioning (SF-12 questionnaire) of 967 men and 812 women (45–83 years) were measured in the CARLA study. We used ordinal logistic regression to examine associations between AGEs, sRAGE, and AGE/sRAGE ratio with physical functioning in sex- and age-stratified models.

**Results:**

Higher levels of AGEs and AGE/sRAGE ratio were associated with lower physical functioning only in women, even after consideration of classical lifestyle and age-related factors (education, BMI, smoking, alcohol consumption, diet, creatinine clearance, diabetes mellitus, lipid lowering and antihypertensive drugs) (odds ratio (OR) =0.86, 95%confidence interval = 0.74–0.98 and OR = 0.86, 95%CI = 0.75–0.98 for AGEs and AGE/sRAGE ratio respectively). We could not demonstrate a significant difference across age.

**Conclusions:**

We showed a sex-specific association between physical functioning and AGEs and AGE/sRAGE, but no stronger associations of the latter with physical functioning. Further investigation is needed in the pathophysiology of this association.

## Background

There is a natural decline in physical functioning with increasing age. This leads to loss of autonomy and eventually need of long-term care. Decline of physical functioning is also associated with multi-morbidity, increased mortality [1], and cognitive impairment [2, 3]. However, the decline in physical functioning has a high inter-individual variability, related to the difference between “biological” and “chronological” age [4]. Advanced glycation end products (AGEs) are considered reliable biomarkers of biological age [5]. AGEs are the irreversible products of the Maillard reaction, a non-enzymatic reaction of reducing sugar with long living proteins and amino acids [6]. They accumulate during normal aging, but also with age-related diseases [7, 8]. A decline in physical functioning has been associated with higher concentrations of AGEs in several studies [9, 10]. Further studies showed the value of AGEs for the prediction of developing disability and severe walking disability [11].

The mechanism of action of AGEs is through non-receptor mediated alterations of protein properties and increase of inflammatory factors by binding to the receptor of AGEs (RAGE). The RAGE is a cell-bound, multi-ligand receptor, which leads to the transcription of pro-inflammatory genes [12]. The sRAGE is the soluble form of RAGE in the blood and has no transmembrane and intracellular domain. sRAGE might be involved in the feedback regulation of the toxic effects of RAGE signaling, hence sRAGE is considered the natural decoy of AGEs [13]. Consistently, several studies suggested that the AGE/sRAGE ratio is stronger than AGEs alone associated with age-related diseases like atherosclerosis [14], endothelial dysfunction [15], hyperthyroidism [16], and end-stage renal disease [17]. Thus, it has been suggested that the ratio is more prone to decline with age than each of the components alone [18]. However, to our knowledge, no study has yet investigated whether this is also true for physical functioning. Moreover, existing studies about the association between AGEs and physical functioning exclusively included participants at advanced age (65 years and above) [9–11, 19]. Therefore, there is a lack of knowledge whether this relationship exists also in younger participants.

Our aim was to assess the association of AGEs, sRAGE, and AGE/sRAGE ratio with physical functioning using data from a population based study in a broad age range (45 to 83 years). We hypothesize that there is variation in the above association across the wider age range (particularly, that the associations might be stronger at older ages). Moreover, we await a stronger association between physical functioning and the AGE/sRAGE ratio than with each of the components alone.

## Methods

### Study population

Cross sectional data from the baseline examination of a total of 1779 participants (967 men and 812 women) of the CARLA study were included in the present analysis. The CARLA-study is a cohort recruited from the general population of the city Halle in Germany [20]. The data collection included a computer-assisted personal standardized interview and medical examinations.

### Outcome: physical functioning (PF)

We used the physical functioning sub-scale of the SF-12 (Medical Outcomes Study Short-Form 12-items [21]) to assess limitations in daily life due to health problems. This subscale consists of two items: impairment in performance of moderate activities and impairment in climbing stairs. The two items had three response categories: “severe limitation”, “minor limitation” and “no limitation”. Following the standard procedures [21], both items were combined to create a physical functioning sub-scale with discrete values between 0 and 100 (0, 25, 50, 75, and 100), where 0 indicates severe limitations and 100 no limitations. Additionally, we conducted analyses with the continuous physical component score (PCS) of the SF-12 questionnaire, which includes information on general health situation, physical functioning, bodily pain, role functioning, mental health, vitality and social functioning [21]. It ranges from 0 to 100, where 0 indicates worst and 100 best health conditions.

### Exposure: AGE and sRAGE measurement

The AGE -specific fluorescence and the sRAGE levels were determined as published before for non-fasting plasma samples of the CARLA cohort participants [22]. Human plasma was thawed, centrifuged at 20,000 g for 3 min at room temperature and diluted 1:20 in PBS (optimal dilution was tested before). One hundred microliter of each diluted sample was transferred to each well of a black 96-well microplate (Greiner, Frickenhausen, Germany). AGE-related fluorescence was measured at least three times on a FLUOstar OPTIMA reader (BMG Labtechnologies, Offenburg, Germany) at 370 +/− 10 nm excitation and 440 +/− 10 nm emission, and the results of the three measurements were averaged. Glucose-modified bovine serum albumin (AGE-BSA) was used for creating an internal standard curve and for plate-to-plate corrections. The results were provided in concentrations equivalent to AGE-BSA. Plasma sRAGE levels were determined using a commercially available enzyme-linked immunosorbent assay ELISA kit (Quantikine; R&D systems) according to the manufacturer’s protocol. Measurements were performed three times and the results were averaged.

### Confounders

Covariates known to affect physical functioning, that were also associated with AGEs were identified from the literature [8, 23–25] and considered as confounders in the analyses. Information about age, years of formal education, smoking status, alcohol consumption, diabetes mellitus (self-reported physician-diagnosed diabetes mellitus or use of antidiabetic medication, ATC-code A10), and osteoporosis (osteoporosis or femoral neck fracture) was acquired by a computer-assisted interview. Information on diet was collected using the paper-version of the EPIC Food Frequency Questionnaire [26]. The use of medication in the previous 7-days was collected through the computer-based IDOM program of the KORA-study (study about the health status of the population in Augsburg, Germany) [27].

Anthropometrical measurements were measured according to standardized protocols. Height and weight were measured using the SECA 220 height measuring system and the SECA 701 digital scale and recorded to the nearest 0.1 cm and 100 g, respectively. BMI was defined as weight measured in kilograms divided by squared-height in meters. Creatinine was determined colorimetrically enzymatically on the Modular system [20]. Creatinine clearance was estimated using the Cockroft-Gault-formula [28].

### Statistical analysis

The AGE/sRAGE ratio was calculated from the raw AGE and sRAGE values. The AGE/sRAGE ratio, AGEs, and sRAGE were log-transformed. In order to facilitate comparison of estimates generated for AGEs, sRAGE and AGE/sRAGE ratio, we normalized the three measurements to units of standard deviation.

We used ordinal logistic regression models to estimate the odds ratio for the association of AGEs, sRAGE and AGE/sRAGE ratio (per one standardized unit increase), and physical functioning (25-points increase in the physical functioning-score). The proportional odds assumption was fulfilled (e.g. fully adjusted model AGEs- physical functioning men χ^2^ = 45.9, *p* = 0.21 and women χ^2^ = 51.2, *p* = 0.09) for the present data. Two models were used with an increasing number of confounders. Model 1 was age-adjusted. To test the additional influence of lifestyle and age-related factors, model 2 was additionally adjusted for years of education, BMI, smoking status, alcohol-consumption, diet, diabetes mellitus, creatinine-clearance, antihypertensive drugs and lipid lowering drugs. Analyses with separate models for lifestyle factors (years of education, BMI, smoking status, alcohol-consumption, diet) and age-related factors (diabetes mellitus, creatinine clearance, antihypertensive drugs, lipid lowering drugs) showed similar results, thus we report only the results of the combined model.

As previous studies had shown sex-specific differences in the effects of AGEs [11, 29], we report the results sex-stratified. For the examination of the stability of the association between physical functioning and laboratory biomarkers across age, we stratified the results for people under the age of 65 and over/ at the age of 65. We used the age of 65 as the cut-point, as all existing studies about AGEs and physical functioning included participants over/at the age of 65. We additionally investigated whether there were differences in age as a continuum or in 10-year age-groups.

Seventy-five participants (3.2%) had missing data for exposure, outcome or confounding variables (see Additional file 1: Table S1). We used the Markov Chain Monte Carlo method with fully conditional specification in SAS for imputation of these data (as implemented in PROC MI) [30]. We generated a database containing 10 imputed datasets. Ordinal logistic regression models were estimated separately for each dataset and results were combined using the MIANALYZE procedure in SAS.

We conducted sensitivity analyses that were restricted to data from participants without missing values (*N* = 930 men/ 792 women) and to participants without extreme values of AGE- and sRAGE (*N* = 951 men; 797 women), defined as the highest 1% of the measurements. A third sensitivity analysis was performed on participants lacking diabetes mellitus or impaired renal function (creatinine clearance < 90 ml/min) as these two diseases strongly increase AGE levels (*N* = 488 men/ 340 women) [7]. We used SAS version 9.4 (SAS Inc., Cary, North Carolina, USA), for the statistical analyses and R version 3.5.0 (R Core Team (2018) for the figures.

## Results

### Study population

Among the 1779 participants of the CARLA study, there were 54% men (967) and 46% women (812), with a mean age of 65 (standard deviation (SD) =10.23) and 64 (SD = 9.94), respectively. Table 1 shows that men had a higher alcohol-consumption, were more likely to smoke, and more often suffered from cardiovascular diseases (myocardial infarction, coronary bypass graft, percutaneously transluminal coronary angioplasty, stroke, and carotid-surgery), while women more often had osteoporosis. No sex differences could be observed for years of education, nutritional status, prevalence of diabetes mellitus, and kidney function (as measured by creatinine clearance).

**Table 1:** Characteristics of the CARLA study population

	Men (*n* = 967)	Women (*n* = 812)
Age, mean ± SD (years)	65 ± 10.23	64 ± 9.94
< 65 years, N (%)	487 (50.36)	455 (56.03)
> = 65 years, N (%)	480 (49.64)	357 (43.97)
BMI, mean ± SD, (kg/m^2^)	28.15 ± 4.08	28.54 ± 5.36
Smoker, N, yes (%)	225 (23.27)	119 (14.66)
Food index^a^, mean ± SD	14.52 ± 3.20	16.44 ± 3.16
Diabetes mellitus, N (%)	154 (15.93)	120 (14.78)
Cardiovascular disease, N (%)	153 (15.82)	48 (5.91)
Osteoporosis, N (%)	64 (6.62)	142 (17.49)
Creatinine clearance, median (P25/P75) (ml/min)	97.22 (76.61/119.41)	88.95 (71.76/109.86)
AGE-levels, median (P25/P75) (relative units)	12,289 (9548/14796)	11,385 (8574/13712)
sRAGE-levels, median (P25/P75) (pg/ml)	827.62 (604.41/1107.88)	964.49 (706.93/1279.58)
AGE/sRAGE, median (P25/P75)	14.46 (10.01/21.09)	11.38 (8.19/16.37)
Limitations in moderate activities, N (%)		
Severe limitation	61 (6.31)	78 (9.61)
Minor limitation	246 (25.44)	305 (37.56)
No limitation	660 (68.25)	429 (52.83)
Limitations in climbing stairs, N (%)		
Severe limitation	69 (7.14)	90 (11.08)
Minor limitation	361 (37.33)	385 (47.41)
No limitation	537 (55.53)	337 (41.50)

Note. *CRP* High sensitive C-reactive protein, *BMI* Body Mass Index, *AGEs* Advanced glycation end products, *sRAGE* Soluble receptor of AGEs, *SD* Standard deviation, *P25/P75* 25th/75th percentile

^a^score 0–30, high score indicates healthier nutrition

Men showed significant higher median levels of plasma-AGEs and AGE/sRAGE ratios than women. In contrast, women had significant higher median levels of plasma-sRAGE. There was no association of AGEs, sRAGE and AGE/sRAGE with age among men and among women (see Additional file 1: Figure S1).

With respect to physical functioning, men reported fewer limitations than women. Almost a third of the men vs. almost half of the women felt at least minor limitations in moderate activities and 44% men vs. 58% women felt limited in climbing stairs. Participants aged 65 years or above were more often slightly or severe limited in moderate activities and climbing stairs than younger participants (Additional file 1: Figure S2).

### Associations between AGEs, sRAGE, and AGE/sRAGE levels and physical functioning

**Fig. 1:**
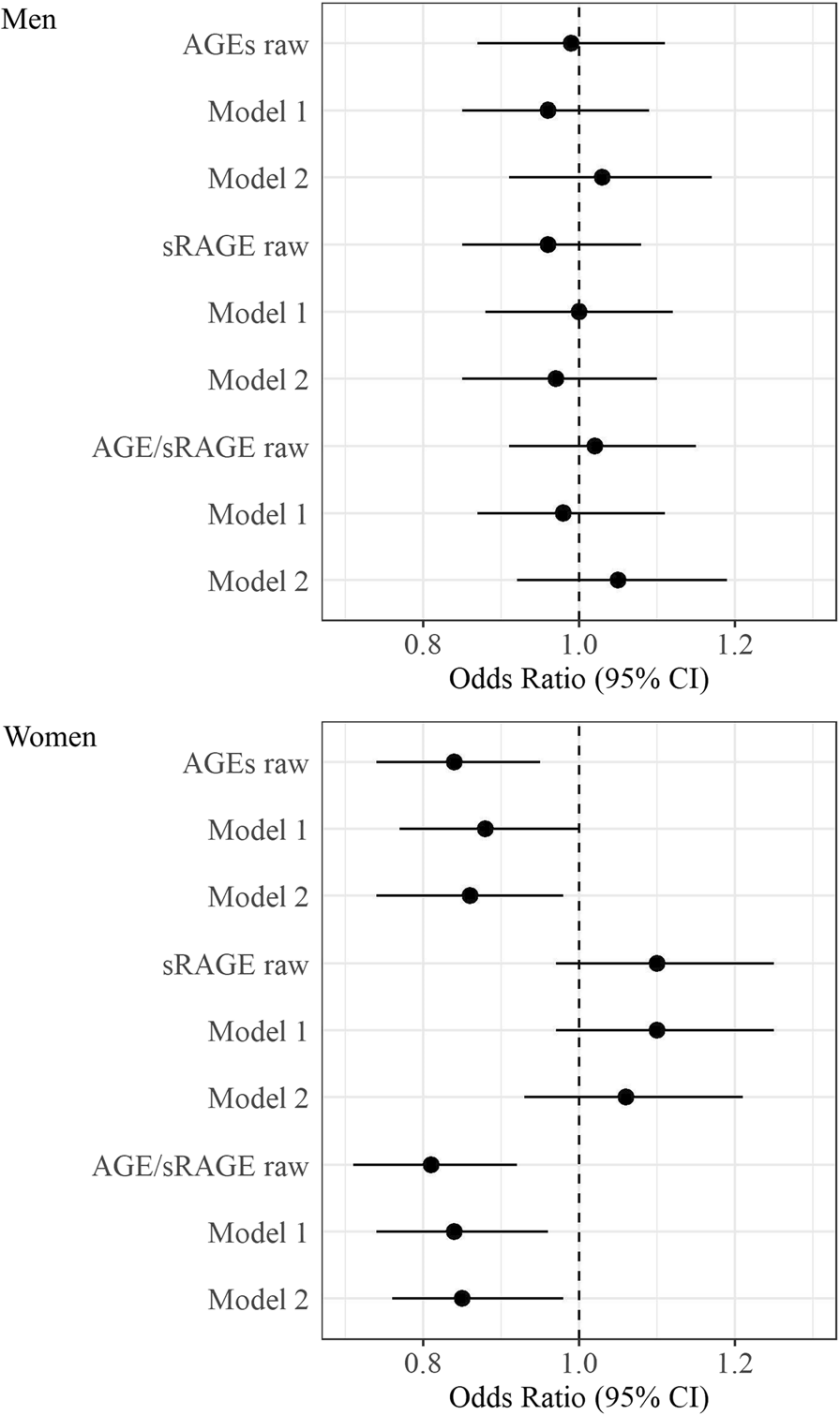
Association between physical functioning and AGEs, sRAGE and AGE/sRAGE for men and women. Ref.: PF = 0 (severe limitations in physical functioning). Model 1: age; Model 2: age, years of education, BMI, alcohol consumption, smoking, diet, diabetes mellitus, creatinine clearance, antihypertensive drugs, lipid lowering drugs. AGEs = advanced glycation end products; sRAGE = soluble receptor of advanced glycation end products; PF = physical functioning; CI = confidence interval

Neither raw nor adjusted ordinal logistic regression models showed significant associations between AGEs, sRAGE, or the AGE/sRAGE ratio and physical functioning for men (Fig. 1 and Additional file 1: Table S2). The odds ratios (OR) ranged from 0.97 (95% confidence interval (CI) 0.85–1.10) for sRAGE to 1.05 (95%CI 0.92–1.19) for the AGE/sRAGE ratio. For women, higher AGE levels were associated with lower physical functioning in the fully adjusted model (OR = 0.86, 95%CI = 0.74–0.98). Similarly, in the fully adjusted model, higher AGE/sRAGE ratios were associated with lower physical functioning (OR = 0.86, 95%CI = 0.75–0.98). Higher levels of sRAGE tended to be associated with higher physical functioning for women (OR = 1.06, 95%CI = 0.93–1.21) (Fig. 1 and Additional file 1: Table S2). There were similar results for AGEs, sRAGE, and AGE/sRAGE in the fully adjusted models in complete case analysis and sensitivity analyses without extreme values (Additional file 1: Figure S3, Table S3, Figure S4 and Table S4), and with the PCS as outcome (Additional file 1: Figure S5 and Table S5). Even the analysis of the subsample without diabetes mellitus and impaired renal function provided similar results (Additional file 1: Figure S6 and Table S6).

### Association between AGEs, sRAGE, and AGE/sRAGE levels and physical functioning in different age groups

**Fig. 2:**
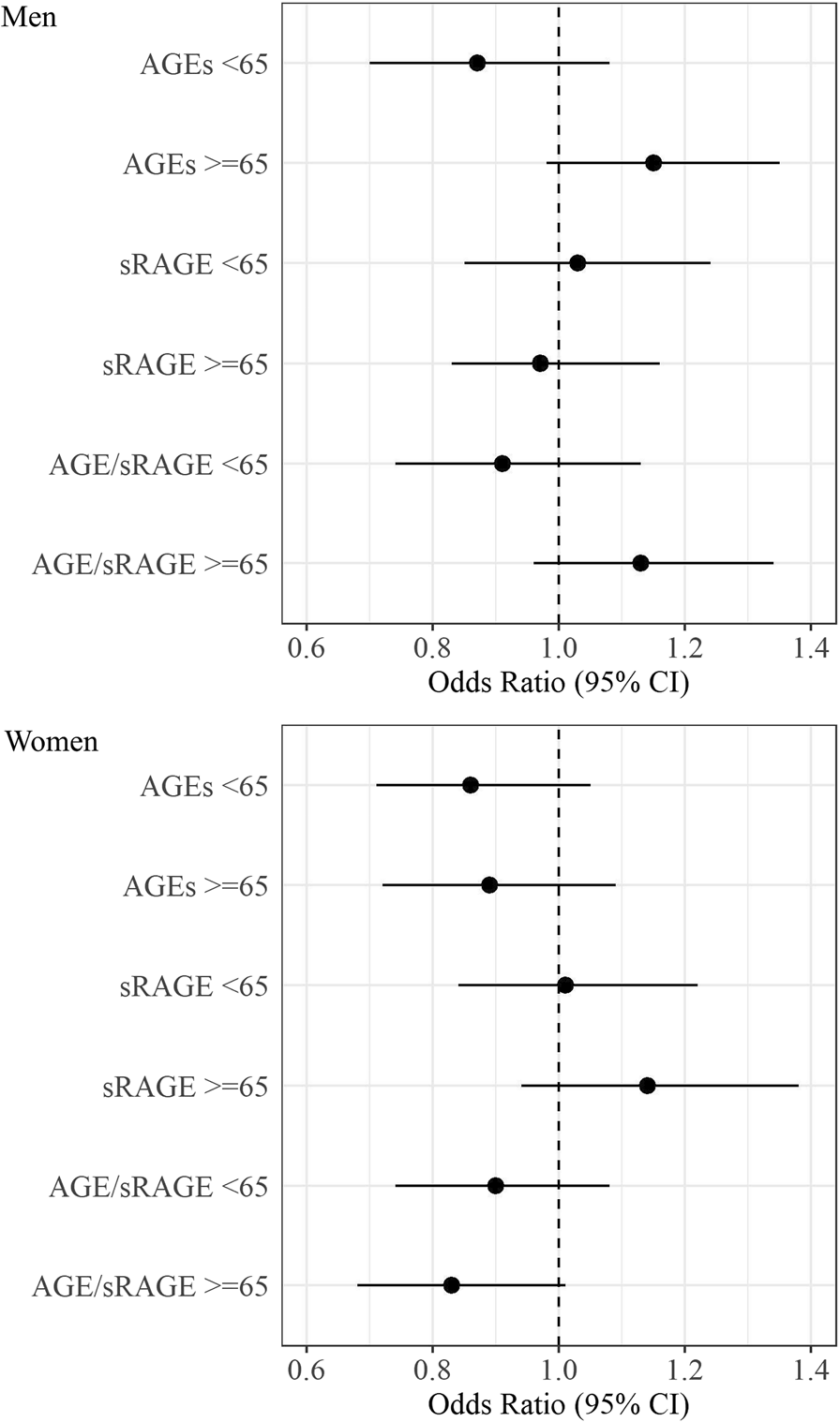
Association between physical functioning and AGEs, sRAGE and AGE/sRAGE for men and women, age-stratified. Age-stratification for men: age < 65 *N* = 487, age > = 65 *N* = 480; and women: age < 65 *N* = 455, age > = 65 *N* = 357. Ref.: PF = 0 (severe limitations in physical functioning). Model 1: age; Model 2: age, years of education, BMI, alcohol consumption, smoking, diet, diabetes mellitus, creatinine clearance, antihypertensive drugs, lipid lowering drugs. AGE = advanced glycation end products; sRAGE = soluble receptor of advanced glycation end products; PF = physical functioning; CI = confidence interval

Figure 2 and Additional file 1: Table S2 show the OR for men and women in the age groups: < 65 years and > =65 years. We saw no significant differences in the associations between the two age groups for men or women. An analysis with 10-years age-groups or age as a continuous variable showed similar results (Additional file 1: Figure S7).

## Discussion

We confirmed results from previous studies that found that higher plasma-AGE values are associated with lower physical functioning [9–11, 19]. However, we found this association only in women, but not in men. Furthermore, we could not confirm the superiority of AGE/sRAGE ratio over AGEs that had been observed for other health outcomes with respect to their association with physical functioning. We could not demonstrate a difference across age groups (Fig. 2). The sex-specific result of our study is consistent with studies in diabetic [29] and non-diabetic [31] middle-aged participants (aged 45 to 64 years), which showed a stronger association of AGEs with mortality in women than in men. It was suggested that AGEs have stronger deleterious effects on participants with a “healthier” life-style and thus a lower risk of cardiovascular diseases, i.e. non-smokers, subjects with low alcohol consumption and higher HDL-cholesterol levels, whereas for participants with an “unhealthy” lifestyle there might be no additional negative effect of AGEs to the classical risk factors (competing risk factors) [29, 31]. This is also true for the CARLA study, where women had a lower risk of several chronic illnesses and a lower prevalence of cardiovascular disease. Although, we adjusted for a number of life-style factors, probably there is residual confounding, like physical activity or housework that cannot be considered and is different between the two sexes. Another explanation could be genetic differences between sexes. Some studies report the association of AGEs with the mechanical properties of bones and their formation process, which could also affect physical functioning [32, 33]. A further study showed a sex-dependent effect of AGEs on vertebral structures and function only in female mice [34]. In our study, we observed higher AGE levels in participants with osteoporosis and higher prevalence of osteoporosis in women (data not shown). However, in regression models, we saw only minor differences in OR and CI when additional adjusting for osteoporosis (Additional file 1: Table S7), possibly due to too low numbers of participants with osteoporosis or residual confounding due to physical activity.

Further, another study investigated the association of AGEs with frailty in an older population (65 years and older) and found an association only in men [11]. They concluded that women at higher risk for frailty and with an unhealthier life-style or with poor health do not participate in cohort studies and thus do not contribute data [11]. Although this selection effect may occur in every cohort, the CARLA cohort is comparable in health-outcomes to the general population reflected in the micro census [35].

It has been hypothesized that the soluble form of the receptor (sRAGE), has a protective effect against the non-receptor and receptor mediated effects of AGEs, which lead to increased muscle stiffening, endothelial dysfunction and inflammation [8, 23, 36]. Therefore, it was supposed that the AGE/sRAGE ratio is more sensitive to age-related decline than each of the components alone [18, 23, 36]. Our results showed that a higher AGE/sRAGE ratio was associated with lower physical functioning in women. To our knowledge no previous study investigated the association between AGE/sRAGE-ratio and physical functioning. However, several studies showed that the AGE/sRAGE-ratio is higher in patients with age-related diseases than in healthy participants. Two studies with cardiac adult and older adult outpatients showed that the severity of atherosclerosis [14] and endothelial dysfunction [15] was associated with a higher AGE/sRAGE-ratio. Additionally, case-control comparisons showed that hyperthyroidism and end-stage renal disease patients had a higher AGE/sRAGE-ratio as compared to healthy adults, whereas there were no differences in levels of AGEs alone [16, 17]. However, in our study the association between the AGEs/sRAGE ratio and physical functioning showed a similar effect as the association of AGEs and physical functioning. Thus, the influence of sRAGE on AGEs doesn’t change the association with physical functioning as much as for chronic diseases. This could be due to the too small effect of endogenous sRAGE on the effect of AGEs. It was hypothesized that the concentrations of endogenous sRAGE are 1000 times lower than needed to act as a sufficient decoy for AGEs [15]. However, in animal models, exogenous administration of sRAGE leads to the suppression of inflammation [37] and attenuation of early acceleration of artherosclerosis [12], showing its role as a possible therapeutic target. Probably higher, exogenous administrated sRAGE levels would influence the association of AGEs to physical functioning.

Further, we evaluated the associations between AGEs, sRAGE and AGE/sRAGE ratio and physical functioning at different life-stages and did not find any difference across age. This finding is interesting as previous studies addressed mainly older participants and extending the evidence to younger age groups appears useful. In women, AGEs are apparently associated with older age, but their association with physical functioning is stable and not restricted to older ages. This supports the notion of AGEs as a marker of biological age, being independent of chronological age – and a potential target of interventions [38].

Our study has several strengths. We included a large number of men and women at a broad age-range from the general population, also including younger age-groups which previously had not been studied before. Additionally, to our knowledge, it is the first study, which calculated the AGE/sRAGE ratio to investigate its association with physical functioning. Moreover, we adjusted our analyses for a variety of important confounders in the association between outcome and exposure. The main limitation of the current study is that we rely on self-reported impairment of physical functioning. Instead of using an objective measurement, such as walking speed or grip strength we measured physical functioning by questions of the SF-12 questionnaire. This might attenuate the strength of association observed in our study. Nevertheless, the SF-12 is a valid questionnaire for measuring physical functioning, reproducing more than 90% of the variance of the long form (SF-36 questionnaire), which sensitively measures physical functioning differences [21, 39, 40]. Moreover, the measurement of plasma-AGEs with fluorescence method could only assess fluorescent AGEs (e.g. pentosidine). However, fluorescence of AGEs correlates also with levels of non-fluorescent AGEs (e.g. N(6)-Carboxymethyllysine) [41] and the results of fluorescence measurement correlate with the results of ELISA measurement of AGEs [42]. Additionally, other fluorescent substances in the plasma could influence the measurement and make it inaccurate. Moreover, the plasma-AGE levels could be strongly influenced by the intake of AGE rich food before the measurement [43]. However, due to the small differences in AGE-levels we observed when stratifying for duration of fasting-time (data not shown), we considered this influence as low.

## Conclusions

In conclusion, this study shows an association between AGEs and AGE/sRAGE ratio and physical functioning only in women. The reasons for the observed sex-differences in the associations still need to be elucidated in further studies. There was no considerable difference in terms of effect size between the association of AGEs, and AGE/sRAGE ratio and physical functioning in contrast to previously suggested better performance of the ratio. We also did not observe differences across age, which supports the notion, that AGEs are a marker of biological rather than chronological age.

## Additional file

**Additional file 1: Table S1.** Number of missing values for variables of interest. **Figure S1.** Scatterplots showing the association between standardied and log-transformed AGEs, sRAGE and AGE/sRAGE with chronological age **Figure S2.** Physical functioning of the study population stratified for sex and age. **Table S2.** Association between physical functioning and log-transformed, standardized AGEs, sRAGE and AGE/sRAGE ratio. **Figure S3.** Association between physical functioning with AGEs, sRAGE and AGE/sRAGE ratio for men and women in complete cases without missings in exposure, outcome or confounding variables. **Table S3.** Complete case analysis. **Figure S4.** Association between physical functioning with AGEs, sRAGE and AGE/sRAGEratio for men and women in a subsample without AGE or sRAGE extreme values (higher 1%). **Table S4.** Analysis without AGE and sRAGE extreme values (higher 1%). **Figure S5.** Association between physical component scale with AGEs, sRAGE and AGE/sRAGE ratio for men and women. **Table S5.** Association between physical component scale and log-transformed, standardized AGEs, sRAGE and AGE/sRAGE ratio. **Figure S6.** Association between physical functioning with AGEs, sRAGE and AGE/sRAGE ratio for men and women in a subsample without diabetes mellitus or impaired renal function. **Table S6.** Association between physical functioning and log-transformed, standardized AGEs, sRAGE and AGE/sRAGE ratio in a subsample without diabetes mellitus and impaired renal function. **Figure S7.** Association between physical functioning with AGEs, sRAGE and AGE/sRAGE ratio for men and women stratified for 10-year age-groups. **Table S7.** Association between physical functioning and log-transformed, standardized AGEs, sRAGE and AGE/sRAGE ratio with and without adjusting for osteoporosis.

## Data Availability

The datasets used and analyzed during the current study are available from the corresponding author (Rafael Mikolajczyk) on reasonable request.

## Abbreviations

AGEs: Advanced glycation end products
CARLA: CARdiovascular disease, Living and Ageing in Halle
CI: Confidence interval
OR: Odds ratio
PCS: Physical component score of SF-12
PF: Physical functioning
SD: Standard deviation
SF-12: Medical Outcomes Study Short-Form 12 items
sRAGE: Soluble receptor for advanced glycation end products
