# Supplementary material for: Advanced glycation end products and their ratio to soluble receptor are associated with limitations in physical functioning only in women: results from the CARLA cohort
Source: BMC Geriatr. 2019 Nov 4;19:299. doi: 10.1186/s12877-019-1323-8 (PMC6829799; doi:10.1186/s12877-019-1323-8)
Supplement: Supplementary file 1 — Additional file 1: Table S1. Number of missing values for variables of interest. Figure S1. Scatterplots showing the association between standardied and log-transformed AGEs, sRAGE and AGE/sRAGE with chronological age Figure S2. Physical functioning of the study population stratified for sex and age. Table S2. Association between physical functioning and log-transformed, standardized AGEs, sRAGE and AGE/sRAGE ratio. Figure S3. Association between physical functioning with AGEs, sRAGE and AGE/sRAGE ratio for men and women in complete cases without missings in exposure, outcome or confounding variables. Table S3. Complete case analysis. Figure S4. Association between physical functioning with AGEs, sRAGE and AGE/sRAGEratio for men and women in a subsample without AGE or sRAGE extreme values (higher 1%). Table S4. Analysis without AGE and sRAGE extreme values (higher 1%). Figure S5. Association between physical component scale with AGEs, sRAGE and AGE/sRAGE ratio for men and women. Table S5. Association between physical component scale and log-transformed, standardized AGEs, sRAGE and AGE/sRAGE ratio. Figure S6. Association between physical functioning with AGEs, sRAGE and AGE/sRAGE ratio for men and women in a subsample without diabetes mellitus or impaired renal function. Table S6. Association between physical functioning and log-transformed, standardized AGEs, sRAGE and AGE/sRAGE ratio in a subsample without diabetes mellitus and impaired renal function. Figure S7. Association between physical functioning with AGEs, sRAGE and AGE/sRAGE ratio for men and women stratified for 10-year age-groups. Table S7. Association between physical functioning and log-transformed, standardized AGEs, sRAGE and AGE/sRAGE ratio with and without adjusting for osteoporosis. [file 12877_2019_1323_MOESM1_ESM.docx]

Table S1. Number of missing values for variables of interest

| Variable | Number of missing values | |
| --- | --- | --- |
|  | men | women |
| AGEs | 9 | 10 |
| sRAGE | 9 | 10 |
| SF-12 Physical functioning item 1 (moderate activities) | 23 | 9 |
| SF-12 Physical functioning item 2 (climbing stairs) | 14 | 7 |
| SF-12 Physical summary scale (PCS) | 55 | 45 |
| Age | 0 | 0 |
| Years of education | 0 | 0 |
| BMI | 0 | 0 |
| Alcohol consumption | 3 | 0 |
| Smoking | 1 | 0 |
| Food-Frequency Index | 2 | 0 |
| Self-reported medical diagnose of diabetes mellitus | 1 | 0 |
| Self-reported medical diagnose of osteoporosis | 6 | 14 |
| Use of antihypertensive drugs | 0 | 0 |
| Use of lipid lowering drugs | 0 | 0 |
| Creatinine-clearance | 7 | 5 |

*Notes. AGEs= advanced glycation end products; sRAGE= soluble receptor of AGEs; BMI= Body mass index*


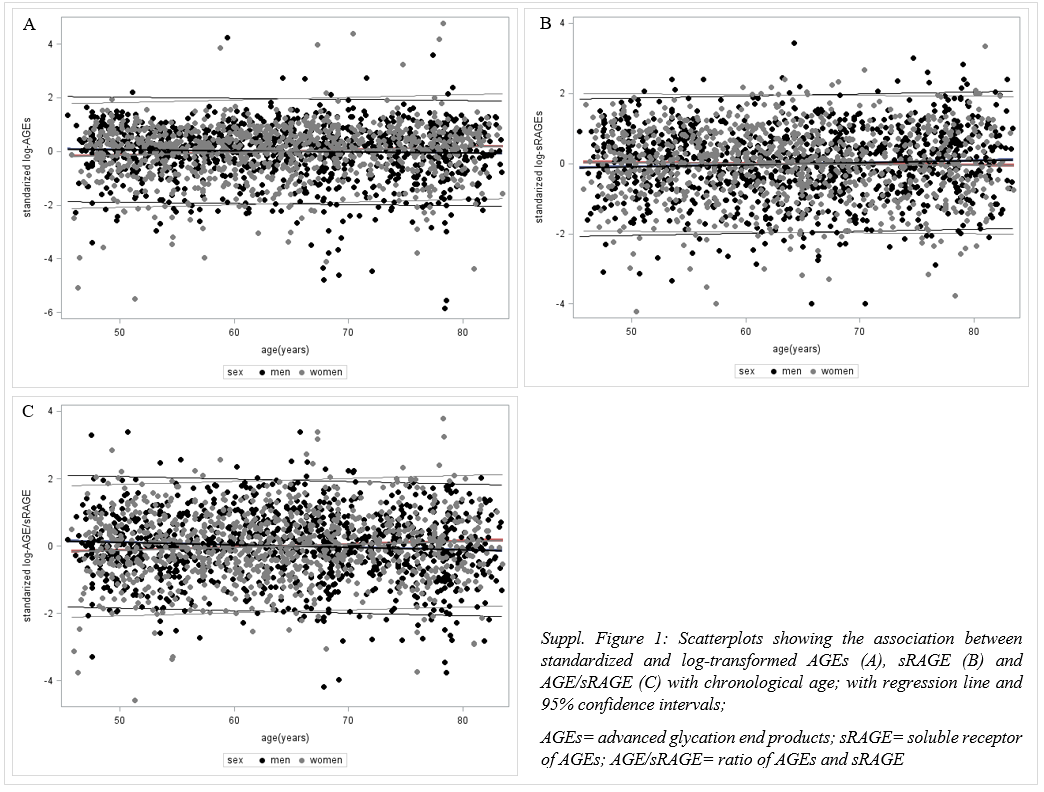


Figure S2: Physical functioning of the study population stratified for sex and age

N= men: 487 <65years; 480 >=65years; women: 455<65years; 357>= 65years

| Table S2. Association between physical functioning (PF) and log-transformed, standardized AGEs, sRAGE and AGE/sRAGE ratio, Odds ratios (95 % confidence interval) | | | | | | |
| --- | --- | --- | --- | --- | --- | --- |
|  | **Raw** | | **Model 1*** | | **Model 2§** | |
|  | *Men* | *Women* | *Men* | *Women* | *Men* | *Women* |
|  | PF 0 = Ref. | | | | | |
| *Association between PF and AGEs* | | | | | | |
| All | 0.99  (0.87; 1.11) | 0.84  (0.74; 0.95) | 0.96  (0.85; 1.09) | 0.88  (0.77; 1.00) | 1.03  (0.91; 1.17) | 0.86  (0.74; 0.98) |
| <65 | 0.87  (0.71; 1.06) | 0.93  (0.78; 1.11) | 0.85  (0.69; 1.05) | 0.95  (0.79; 1.13) | 0.87  (0.70; 1.08) | 0.86  (0.71; 1.05) |
| >= 65 | 1.03  (0.88; 1.20) | 0.82  (0.67; 0.99) | 1.04  (0.89; 1.21) | 0.80  (0.66; 0.97) | 1.15  (0.98; 1.35) | 0.89  (0.72; 1.09) |
| *Association between PF and sRAGE* | | | | | | |
| All | 0.96  (0.85; 1.08) | 1.10  (0.97; 1.25) | 1.00  (0.88; 1.12) | 1.10  (0.97; 1.25) | 0.97  (0.85; 1.10) | 1.06  (0.93; 1.21) |
| <65 | 1.08  (0.90; 1.30) | 1.09  (0.91;1.30) | 1.05  (0.88; 1.27) | 1.11  (0.93; 1.32) | 1.03  (0.85; 1.24) | 1.01  (0.84; 1.22) |
| >= 65 | 0.91  (0.78; 1.07) | 1.12  (0.93; 1.35) | 0.98  (0.83; 1.16) | 1.09  (0.91; 1.32) | 0.97  (0.82; 1.16) | 1.14  (0.94; 1.38) |
| *Association between PF and AGE/ sRAGE* | | | | | | |
| All | 1.02  (0.91; 1.15) | 0.81  (0.71; 0.92) | 0.98  (0.87; 1.11) | 0.84  (0.74; 0.96) | 1.05  (0.92; 1.19) | 0.86  (0.75; 0.98) |
| <65 | 0.86  (0.71; 1.04) | 0.89  (0.74; 1.06) | 0.87  (0.72; 1.05) | 0.88  (0.74; 1.06) | 0.91  (0.74; 1.11) | 0.90  (0.74; 1.08) |
| >= 65 | 1.09  (0.94; 1.28) | 0.79  (0.65; 0.95) | 1.05  (0.89; 1.23) | 0.79  (0.65; 0.96) | 1.13  (0.96; 1.34) | 0.83  (0.68; 1.01) |
| *Notes: * Model 1: age; § Model 2: age, years of education ,BMI, alcohol consumption, smoking, diet, diabetes mellitus, creatinine clearance, antihypertensive drugs, lipid lowering drugs;*  *N= 967 men; 812 women;*  *AGEs= advanced glycation end products; sRAGE= soluble receptor of AGEs; AGE/sRAGE: ratio of AGE and sRAGE; PF= total score physical functioning (SF-12)* | | | | | | |


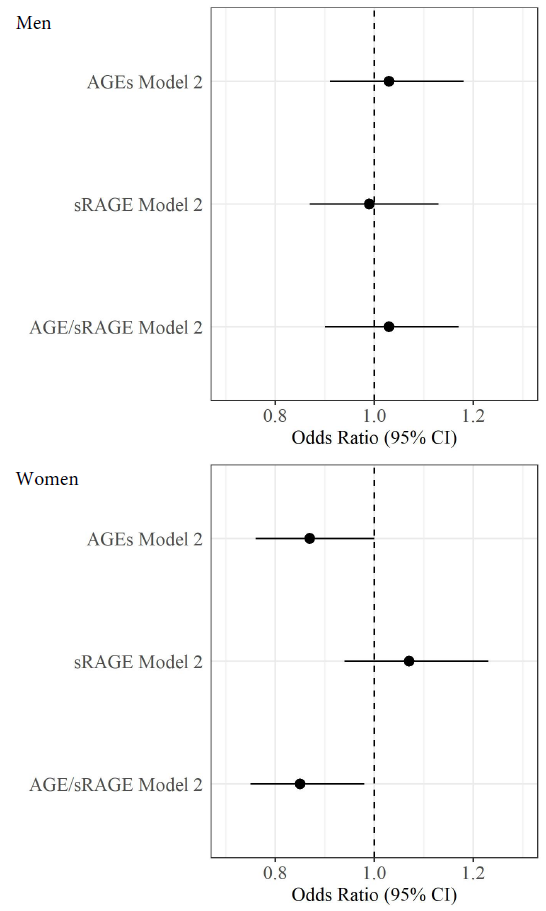


Figure S3: Association between physical functioning (PF) with advanced glycation end products (AGEs), soluble receptor of AGEs (sRAGE) and ratio of AGEs and sRAGE (AGE/sRAGE) for men (N=930) and women (N=792) in **complete cases** without missings in exposure, outcome or confounding variables; Odds ratios (95 % confidence interval)

Ref.: PF=0 (severe limitations in physical functioning)

Model 2: age, years of education, BMI, alcohol consumption, smoking, diet, diabetes mellitus, creatinine clearance, antihypertensive drugs, lipid lowering drugs;

CI= confidence interval

| Table S3. Complete case analysis;  Association between physical functioning (PF) and log-transformed, standardized AGEs, sRAGE and AGE/sRAGE ratio, Odds ratios (95 % confidence interval) | | | | | | |
| --- | --- | --- | --- | --- | --- | --- |
|  | **Raw** | | **Model 1*** | | **Model 2§** | |
|  | *Men* | *Women* | *Men* | *Women* | *Men* | *Women* |
|  | PF 0 = Ref. | | | | | |
| *Association between PF and AGEs* | | | | | | |
| All | 0.98  (0.87; 1.11) | 0.84  (0.74; 0.96) | 0.96  (0.85; 1.09) | 0.88  (0.77; 1.01) | 1.03  (0.91; 1.18) | 0.87  (0.76; 1.00) |
| *Association between PF and sRAGE* | | | | | | |
| All | 0.97  (0.86; 1.10) | 1.12  (0.98; 1.26) | 1.01  (0.90; 1.15) | 1.11  (0.97; 1.26) | 0.99  (0.87; 1.13) | 1.07  (0.94; 1.23) |
| *Association between PF and AGE/sRAGE* | | | | | | |
| All | 1.01  (0.89; 1.14) | 0.81  (0.71; 0.92) | 0.96  (0.85; 1.09) | 0.84  (0.74; 0.96) | 1.03  (0.90; 1.17) | 0.85  (0.75; 0.98) |
| *Notes: * Model 1: age; § Model 2: age, years of education ,BMI, alcohol consumption, smoking, diet , diabetes mellitus, creatinine clearance, antihypertensive drugs, lipid lowering drugs;*  *N=930 men; 792 women*  *AGEs= advanced glycation end products; sRAGE= soluble receptor of AGEs; AGE/sRAGE: ratio of AGE and sRAGE; PF= total score physical functioning (SF-12)* | | | | | | |


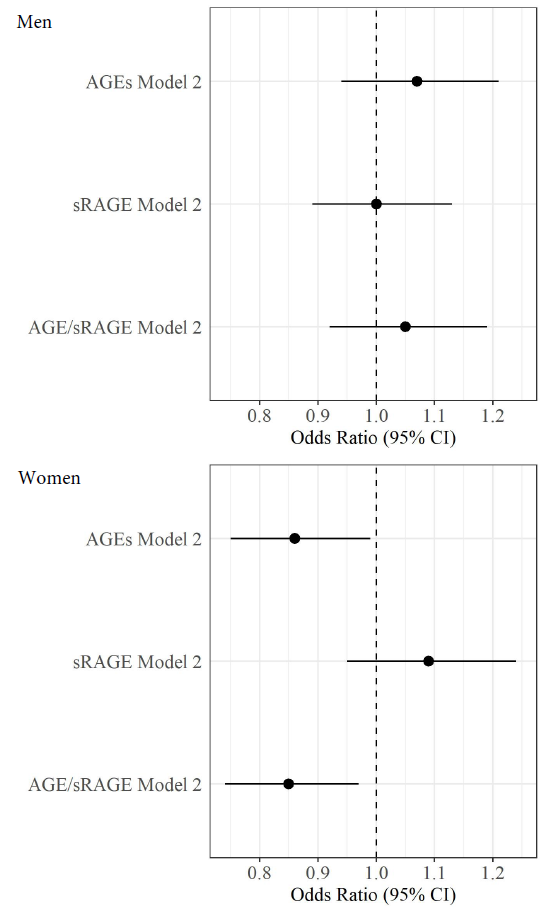


Figure S4: Association between physical functioning (PF) with advanced glycation end products (AGEs), soluble receptor of AGEs (sRAGE) and ratio of AGEs and sRAGE (AGE/sRAGE) for men (N=951) and women (N=797) in a subsample **without AGE or sRAGE extreme values** (higher 1%); Odds ratios (95 % confidence interval)

Ref.: PF=0 (severe limitations in physical functioning)

Model 2: age, years of education, BMI, alcohol consumption, smoking, diet, diabetes mellitus, creatinine clearance, antihypertensive drugs, lipid lowering drugs;

CI= confidence interval

| Table S4. Analysis without AGE and sRAGE extreme values (higher 1%)  Association between physical functioning (PF) and log-transformed, standardized AGEs, sRAGE and AGE/sRAGE ratio, Odds ratios (95 % confidence interval) | | | | | |
| --- | --- | --- | --- | --- | --- |
| Raw | | **Model 1*** | | **Model 2§** | |
| *Men* | *Women* | *Men* | *Women* | *Men* | *Women* |
| PF 0 = Ref. | | | | | |
| *Association between PF and AGEs* | | | | | |
| 1.05  (0.93; 1.19) | 0.86  (0.76; 0.98) | 1.03  (0.91; 1.16) | 0.89  (0.78; 1.01) | 1.07  (0.94; 1.21) | 0.86  (0.75; 0.99) |
| *Association between PF and sRAGE* | | | | | |
| 1.02  (0.91; 1.15) | 1.14  (1.00; 1.29) | 1.06  (0.93; 1.19) | 1.12  (0.98; 1.27) | 1.00  (0.88; 1.13) | 1.09  (0.95; 1.24) |
| *Association between PF and AGE/ sRAGE* | | | | | |
| 1.02  (0.90; 1.15) | 0.81  (0.71; 0.92) | 0.98  (0.86; 1.10) | 0.84  (0.73; 0.95) | 1.05  (0.92; 1.19) | 0.85  (0.74; 0.97) |
| *Notes: * Model 1: age; § Model 2: age, years of education ,BMI, alcohol consumption, smoking, diet, diabetes mellitus, creatinine clearance, antihypertensive drugs, lipid lowering drugs;*  *N=951 men; 797 women*  *AGEs= advanced glycation end products; sRAGE= soluble receptor of AGEs; AGE/sRAGE: ratio of AGE and sRAGE; PF= total score physical functioning (SF-12)* | | | | | |


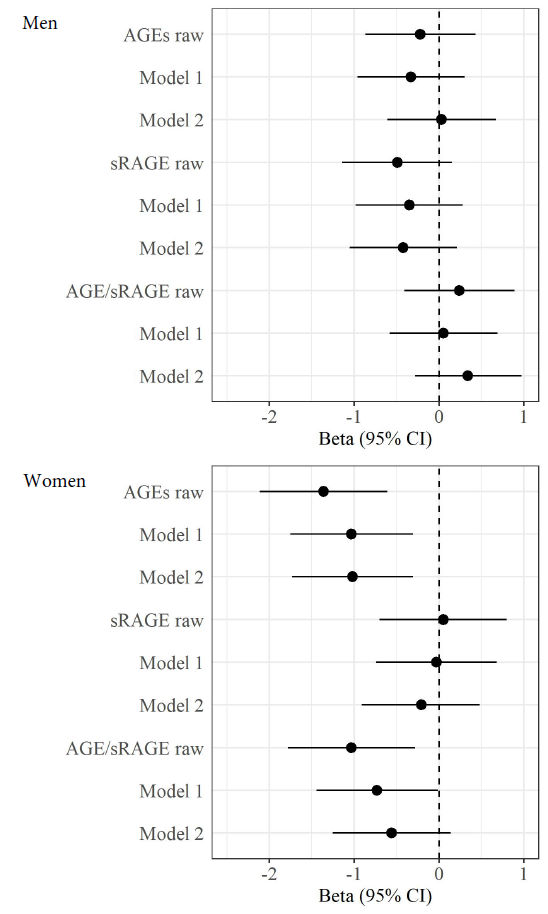


Figure S5: Association between **physical component scale** (PCS) with advanced glycation end products (AGEs), soluble receptor of AGEs (sRAGE) and ratio of AGEs and sRAGE (AGE/sRAGE) for men (N=967) and women (N=812), Beta (β) (95 % confidence interval)

Model 2: age, years of education, BMI, alcohol consumption, smoking, diet, diabetes mellitus, creatinine clearance, antihypertensive drugs, lipid lowering drugs;

CI= confidence interval

| Table S5. Association between physical component scale (PCS) and log-transformed, standardized AGEs, sRAGE and AGE/sRAGE ratio, β (95 % confidence interval) | | | | | |
| --- | --- | --- | --- | --- | --- |
| Raw | | Model 1* | | Model 2§ | |
| *Men* | *Women* | *Men* | *Women* | *Men* | *Women* |
| *Association between PCS and AGEs* | | | | | |
| 0.22  ( -0.87; 0.43) | -1.36  ( -2.11; -0.61) | -0.33  ( -0.96; 0.30) | -1.03  ( -1.75; 0.31) | 0.03  ( -0.61; 0.67) | -1.02  ( -1.73; -0.31) |
| *Association between PCS and sRAGE* | | | | | |
| -0.49  ( -1.14; 0.15) | 0.05  ( -0.70; 0.80) | -0.35  ( -0.98; 0.28) | -0.03  ( -0.74; 0.68) | -0.42  ( -1.05; 0.21) | -0.21  ( -0.91; 0.48) |
| *Association between PCS and AGE/ sRAGE* | | | | | |
| 0.24  ( -0.41; 0.89) | -1.03  ( -1.78; -0.28) | 0.05  ( -0.58; 0.69) | -0.73  ( -1.44; 0.01) | 0.34  ( -0.28; 0.97) | -0.56  ( -1.25; 0.14) |
| *Notes: * Model 1: age; § Model 2: age, years of education, BMI, alcohol consumption, smoking, diet , diabetes mellitus, creatinine clearance, antihypertensive drugs, lipid lowering drugs;*  *N= 967 men; 812 women*  *AGEs= advanced glycation end oroducts; sRAGE= soluble receptor of AGEs; AGE/sRAGE: ratio of AGE and sRAGE; PCS = physical sum scale (SF-12).* | | | | | |


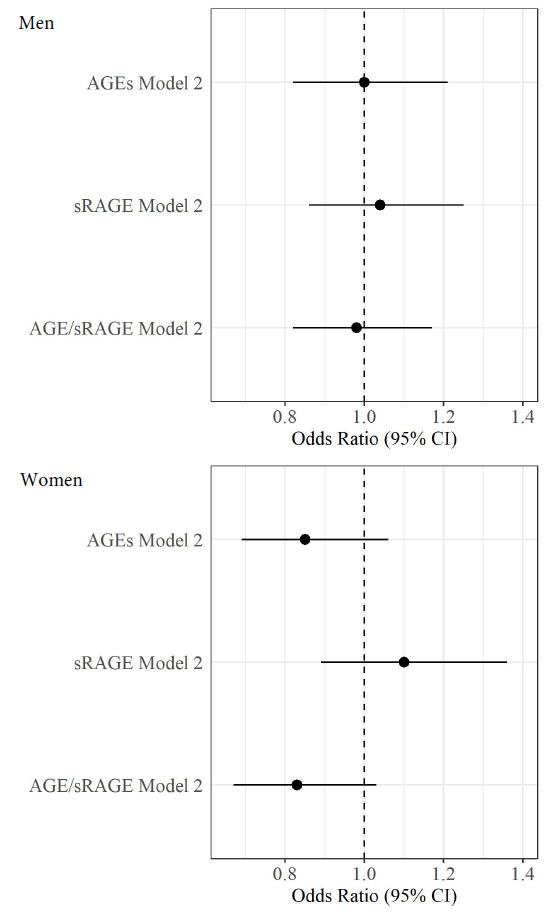


Figure S6: Association between physical functioning (PF) with advanced glycation end products (AGEs), soluble receptor of AGEs (sRAGE) and ratio of AGEs and sRAGE (AGE/sRAGE) for men (N=488) and women (N=340) in a **subsample without diabetes mellitus or impaired renal function**, Odds ratios (95 % confidence interval)

Ref.: PF=0 (severe limitations in physical functioning)

Model 2: age, years of education, BMI, alcohol consumption, smoking, diet, creatinine clearance, antihypertensive drugs, lipid lowering drugs;

CI= confidence interval

| Table S6. Association between physical functioning (PF) and log-transformed, standardized AGEs, sRAGE and AGE/sRAGE ratio in a subsample without diabetes mellitus and impaired renal function, Odds ratios (95 % confidence interval) | | | | | |
| --- | --- | --- | --- | --- | --- |
| Raw | | **Model 1*** | | **Model 2§** | |
| *Men* | *Women* | *Men* | *Women* | *Men* | *Women* |
| PF 0 = Ref. | | | | | |
| *Association between PF and AGEs* | | | | | |
| 0.98  (0.82; 1.17) | 0.94  (0.77; 1.14) | 0.96  (0.80; 1.15) | 0.94  (0.77; 1.15) | 1.00  (0.82; 1.21) | 0.85  (0.69; 1.06) |
| *Association between PF and sRAGE* | | | | | |
| 1.07  (0.89; 1.28) | 1.16  (0.95; 1.41) | 1.04  (0.87; 1.25) | 1.15  (0.94; 1.40) | 1.04  (0.86; 1.25) | 1.10  (0.89; 1.36) |
| *Association between PF and AGE/ sRAGE* | | | | | |
| 0.94  (0.79; 1.12) | 0.85  (0.70; 1.04) | 0.94  (0.79; 1.13) | 0.86  (0.70; 1.05) | 0.98  (0.82; 1.17) | 0.83  (0.67; 1.03) |
| *Notes: * Model 1: age; § Model 2: age, years of education ,BMI, alcohol consumption, smoking, diet , creatinine clearance, antihypertensive drugs, lipid lowering drugs;*  *N=488 men; 340 women*  *AGEs= advanced glycation end products; sRAGE= soluble receptor of AGEs; AGE/sRAGE: ratio of AGE and sRAGE; PF= total score physical functioning (SF-12).* | | | | | |


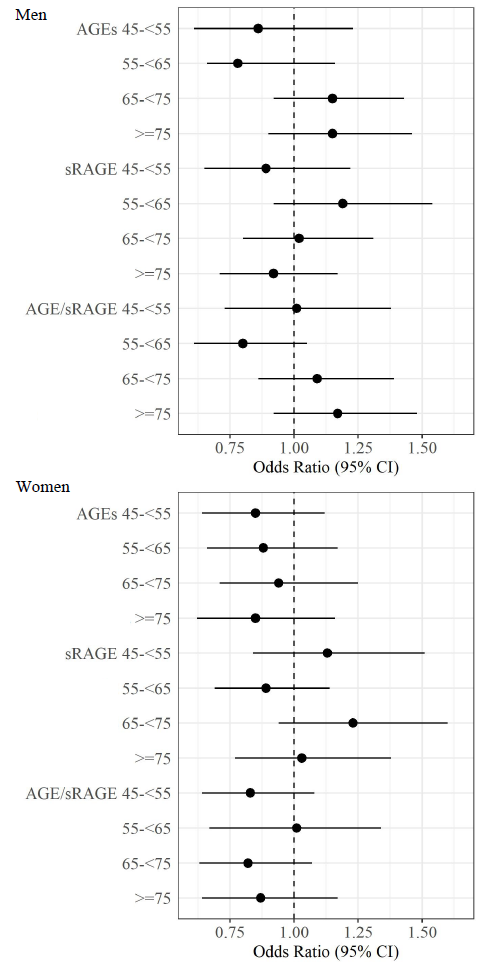


Figure S7: Association between physical functioning (PF) with advanced glycation end products (AGEs), soluble receptor of AGEs (sRAGE) and ratio of AGEs and sRAGE (AGE/sRAGE) for men (N=976) and women (N=812) **stratified for 10-year age-groups**, Odds ratios (95 % confidence interval)

*Ref.: PF=0 (severe limitations in physical functioning)*

*Adjusted for: age, years of education, BMI, alcohol consumption, smoking, diet, creatinine clearance, antihypertensive drugs, lipid lowering drugs (Model 2);*

*CI= confidence interval*

| Table S7. Association between physical functioning (PF) and log-transformed, standardized AGEs, sRAGE and AGE/sRAGE ratio with and without adjusting for osteoporosis, Odds ratios (95 % confidence interval) | | | | |
| --- | --- | --- | --- | --- |
|  | **Model 2§** | | **Model 2a** | |
|  | *Men* | *Women* | *Men* | *Women* |
| PF 0 = Ref. | | | | |
| *Association between PF and AGEs* | | | | |
| All | 1.03  (0.91; 1.17) | 0.86  (0.74; 0.98) | 1.04  (0.92; 1.18) | 0.87  (0.75; 1.00) |
| <65 | 0.87  (0.70; 1.08) | 0.86  (0.71; 1.05) | 0.85  (0.69; 1.06) | 0.88  (0.72; 1.07) |
| >= 65 | 1.15  (0.98; 1.35) | 0.89  (0.72; 1.09) | 1.14  (0.97; 1.34) | 0.88  (0.71; 1.08) |
| *Association between PF and sRAGE* | | | | |
| All | 0.97  (0.85; 1.10) | 1.06  (0.93; 1.21) | 0.97  (0.85; 1.10) | 1.06  (0.93; 1.22) |
| <65 | 1.03  (0.85; 1.24) | 1.01  (0.84; 1.22) | 1.01  (0.83; 1.22) | 1.01  (0.81; 1.19) |
| >= 65 | 0.97  (0.82; 1.16) | 1.14  (0.94; 1.38) | 0.97  (0.82; 1.16) | 1.14  (0.94; 1.39) |
| *Association between PF and AGE/ sRAGE* | | | | |
| All | 1.05  (0.92; 1.19) | 0.86  (0.75; 0.98) | 1.06  (0.93; 1.20) | 0.86  (0.75; 0.99) |
| <65 | 0.91  (0.74; 1.11) | 0.90  (0.74; 1.08) | 0.91  (0.74; 1.12) | 0.91  (0.75; 1.10) |
| >= 65 | 1.13  (0.96; 1.34) | 0.83  (0.68; 1.01) | 1.14  (0.97; 1.35) | 0.83  (0.68; 1.01) |
| *Notes: § Model 2: age, years of education ,BMI, alcohol consumption, smoking, diet , diabetes mellitus, creatinine clearance, antihypertensive drugs, lipid lowering drugs; Model 2a: Model 2 + osteoporosis as confounder*  *N= 967 men; 812 women*  *AGEs= advanced glycation end products; sRAGE= soluble receptor of AGEs; AGE/sRAGE: ratio of AGE and sRAGE; PF= total score physical functioning (SF-12).* | | | | |
